# Two novel mutations of fibrillin-1 gene correlate with different phenotypes of Marfan syndrome in Chinese families

**Published:** 2013-04-05

**Authors:** Feng Zhao, Xinyuan Pan, Kanxing Zhao, Chen Zhao

**Affiliations:** 1 Department of Cardiology and Surgery, Tianjin Chest and Heart Hospital, Tianjin Medical University, Tianjin, China; 2 The First Affiliated Hospital with Nanjing Medical University, State Key Laboratory of Reproductive Medicine, Nanjing, China; 3 Tianjin Eye Hospital, Tianjin Key Laboratory of Ophthalmology and Visual Science, Tianjin Medical University, Tianjin, China

## Abstract

**Purpose:**

To identify the causative mutations in two Chinese families with autosomal dominant Marfan syndrome and to describe the associated phenotypes.

**Methods:**

Complete physical, ophthalmic, and cardiovascular examinations were given to the patients and unaffected individuals in the two families. Exclusive linkage mapping was performed for transforming growth factor beta receptor II (TGFBR2) and fibrillin-1 (*FBN1*) loci in both families. The entire coding region and flanking splice sites of the *FBN1* gene were screened for mutations in the two families with Sanger sequencing. The potential mutations of *FBN1* were tested in 100 normal controls.

**Results:**

Lens dislocation was observed in two out of ten patients in the MF1 family and all patients in the MF2 family. However, the MF1 family displayed more severe cardiovascular and skeletal system involvement compared with the MF2 family. The transforming growth factor beta receptor II locus was excluded in both families by linkage analysis. A maximum multipoint lod score score of 2.83 was obtained for marker D15S992 (located in the *FBN1* gene) in the MF1 family and 1.51 for the same marker in the MF2 family. Two novel mutations of *FBN1*, p.C271* and p.C637Y, were identified in the MF1 and MF2 families, respectively.

**Conclusions:**

Genotype-phenotype correlations in this study indicate that nonsense mutations of *FBN1* may correlate with relatively severe systemic phenotypes when compared with cysteine substitutions, the most common type of *FBN1* mutations. Genetic diagnosis for patients with Marfan syndrome would help with genetic counseling, clinical intervention, and prognosis.

## Introduction

Marfan syndrome (MFS; MIM #154700) is a pleiotropic, autosomal dominant inherited disorder of connective tissue with an estimated prevalence of up to 1:5,000 [1]. MFS is characterized by variable phenotypic manifestations mainly in cardiovascular, ocular, and skeletal systems. Thus far, two causative genes have been demonstrated for MFS: fibrillin-1 (*FBN1*) and transforming growth factor beta receptor II (*TGFBR2*). Approximately 90% of MFS cases are caused by mutations in the *FBN1* gene (15q21.1) [2]; whereas a second MFS causative gene, the *TGFBR2* (3p22) gene, was identified in a French family with MFS originally found not to be associated with *FBN1* in 2004 [3].

Fibrillin-1 is the major constitutive element of extracellular microfibrils encoded by the large *FBN1* gene that contains 66 exons spanning 235 kb of genomic DNA on chromosome 15q21.1. Fibrillin-1 is a 350-kDa glycoprotein and has a modular structure comprising 47 epidermal growth factor-like (EGF) domains, seven transforming growth factor β1-binding protein-like (TB) domains, a proline-rich region, hybrid modules, and unique N- and C-termini. EGF modules are approximately 45 residues long and are characterized by six conserved cysteine residues that form three intramodule disulfide bonds [4,5]. Of the 47 EGF domains, 43 contain a consensus cysteine-rich sequence for calcium binding (cb-EGF), which is predicted to play an important role in microfibril stability and assembly [6]. All cysteines in fibrillin-1 are evolutionarily conserved, emphasizing their essential function. Fibrillin-1 mutations break microfibril formation, weaken the connective tissue, and result in fibrillin protein abnormalities [7].

In this study, we performed exclusive linkage analysis on two families with MFS with distinct phenotypes by using microsatellite markers spanning the critical regions of the two known MFS loci. Consistent with the positive linkage found on the *FBN1* locus in the two families, we identified two novel *FBN1* mutations, each of which correlated with distinct phenotypes presented by the corresponding pedigree.

## Methods

### Patients and controls

**Figure 1:**
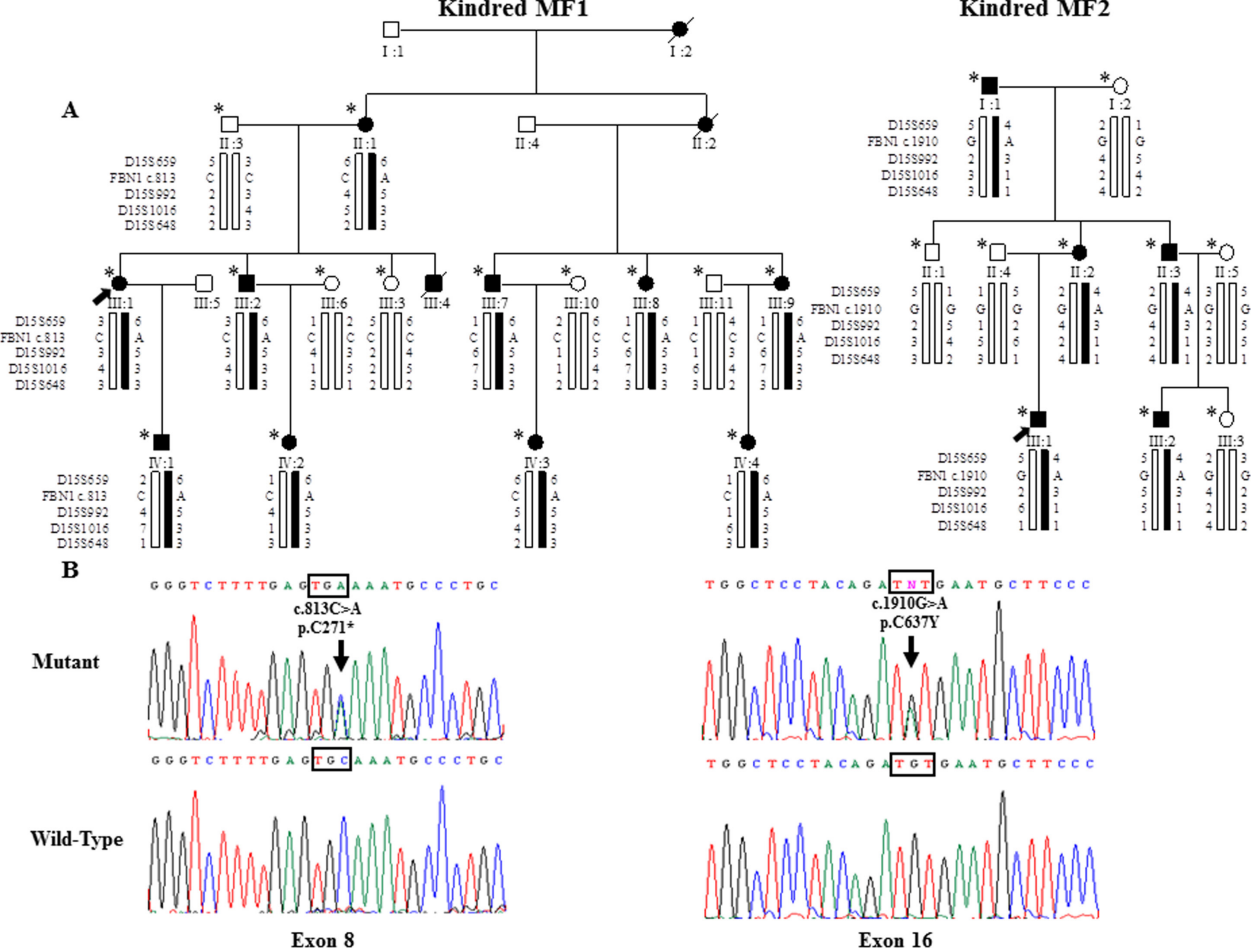
Identification of two *FBN1* mutations in two Chinese families (MF1 and MF2) with MFS. **A**: In the pedigrees of MF1 and MF2, men and women are symbolized by squares and circles, and affected and unaffected members are represented by filled and open symbols, respectively. A diagonal line through the pedigree symbol indicates a deceased individual, and an arrow indicates the proband. The asterisk at the upper left of the pedigree symbol indicates participation in the study. Haplotypes for the *FBN1* locus are shown, with filled boxes representing affected haplotypes and white boxes indicating unaffected haplotypes. **B**: Sequence chromatograms from normal (wild-type) and affected (mutant) members in kindreds MF1 and MF2 are shown. Arrows indicate the locations of the point mutations.

Fifteen patients (ten from kindred MF1 and five from kindred MF2) were recruited in Tianjin Chest and Heart Hospital and Tianjin Eye Hospital. The fifteen patients were clinically diagnosed as MFS at the time of recruitment (Figure 1). The patients’ gender and ages are shown in the Table 1.Eleven individuals in kindred MF1 (ten affected members and one unaffected sibling) and seven individuals in kindred MF2 (five affected members and two presently unaffected siblings) underwent ophthalmic examinations, including assessment of best-corrected visual acuity, slit-lamp and direct fundus examinations, and systemic evaluations including physical examinations, echocardiography with measurement of the aortic root diameter, assessment of valve morphology and function, skeletal features, and skin extensibility.

**Table 1:** Clinical features of patients from MF1 and MF2 families

Family	MF1	MF1	MF1	MF1	MF1	MF1	MF1	MF1	MF1	MF1	MF2	MF2	MF2	MF2	MF2
Individual	II:1	III:1	III:2	III:7	III:8	III:9	IV:1	IV:2	IV:3	IV:4	I:1	II:2	II:3	III:1	III:2
Sex/Age (yrs)	F/61	F/39	M/36	M/37	F/34	F/32	M/17	F/15	F/15	F/10	M/57	F/35	M/32	M/13	M/9
**Cardiovascular system**															
Aortic root dimension(mm)	58	65	62	60	52	38	28	27	35	25	36	25	24	21	20
Mitral valve prolapse	(-)	(+)	(-)	(-)	(+)	(+)	(-)	(-)	(-)	(-)	(-)	(-)	(-)	(-)	(-)
**Ocular system**															
lens dislocation	(-)	(+)	(-)	(+)	(-)	(-)	(-)	(-)	(-)	(-)	(+)	(+)	(+)	(+)	(+)
myopia	(-)	(+)	(-)	(-)	(-)	(-)	(-)	(-)	(-)	(-)	(+)	(+)	(-)	(-)	(-)
Strabismus	(-)	(-)	(-)	(-)	(-)	(-)	(-)	(-)	(-)	(-)	(+)	(-)	(-)	(-)	(-)
Glaucoma	(-)	(-)	(-)	(-)	(-)	(-)	(-)	(-)	(-)	(-)	(-)	(+)	(+)	(-)	(-)
**Skeletal system**															
Height (cm)	184	190	187	188	185	187	188	180	176	140	187	180	185	173	139
Arm span (cm)	192	191	186	188	190	189	190	180	173	138	189	181	187	170	137
Pectys deformities	(+)	(+)	(+)	(+)	(-)	(-)	(-)	(+)	(-)	(-)	(-)	(-)	(-)	(-)	(-)
Scoliosis	(+)	(+)	(-)	(+)	(+)	(+)	(+)	(+)	(-)	(-)	(-)	(-)	(-)	(-)	(-)
Joint hypermobility	(-)	(+)	(+)	(+)	(-)	(-)	(+)	(-)	(+)	(+)	(-)	(-)	(-)	(+)	(-)
**Other manifestations**															
Hyperextensible skin	(-)	(-)	(+)	(+)	(-)	(+)	(-)	(-)	(-)	(-)	(-)	(-)	(-)	(-)	(-)
Striae	(+)	(+)	(-)	(+)	(+)	(+)	(-)	(+)	(-)	(-)	(+)	(+)	(+)	(-)	(-)

F, female; M, male.

Peripheral blood samples were drawn from median cubital vein from 15 individuals of kindred MF1, including ten affected members, one unaffected member, and four spouses, and ten individuals of kindred MF2, including five affected members, two unaffected siblings, and three spouses (Figure 1A). In addition, peripheral blood samples were collected from 100 normal Chinese volunteers with neither history of MFS nor related fibrillinopathy and were used as normal controls in the mutation study. All the peripheral blood samples were stored at -80°C before extraction of genomic DNA. Informed consent was obtained from all cases for sample collection and molecular analysis, and human studies were prospectively reviewed and approved by local institutional ethical review boards according to the Declaration of Helsinki. Genomic DNA was isolated from peripheral leukocytes.

### Genotyping of microsatellite markers and linkage analysis

Exclusive linkage analyses were performed on the two families by genotyping microsatellite markers spanning two known loci for MFS: *FBN1* (D15S659, D15S992, D15S1016, and D15S648) and *TGFBR2* (D3S2336, D3S2335, D3S1283, D3S3727, and D3S2432). Amplification of these microsatellite markers with PCR was performed using primers labeled with FAN, TET, or HEX and experimental conditions as previously described [8]. Briefly, the PCR program consists of an initial denaturation for 10 min at 95°C, followed by 30 cycles of 30 s at 95°C, 30 s at 55°C and 60 s at 72°C, and a final extension at 72°C for 7 min. The polymerase chain reactions were carried out in 10μl reactions containing 10 ng genomic DNA, 1xPCR buffer, 200 μM of each deoxyribonucleoside triphosphate (dNTP), 2.5 mM MgCl_2_, 0.15 μM of each primer (labelled with Fam, Tet or Hex) and 0.5 U of AmpliTaq Gold DNA polymerase (Applied Biosystems). The PCR products were appropriately pooled according to allele size and labeling, mixed with GeneScan 500 TAMRA standard (Applied Biosystems, Foster City, CA), denatured, loaded onto 6% standard denaturing polyacrylamide gels, and run in the ABI 377XL sequencer (Applied Biosystems) for fluorescent detection. Genotyping data were collected using GeneScan Analysis 3.1 (PerkinElmer Inc., Waltham, MA) and analyzed using the Genotyper 2.0 software package (PerkinElmer).

Linkage analyses were performed by calculating multipoint lod scores using the LINKAGE software package Simwalk2, Version 3.35. MFS in both families was modeled as an autosomal dominant trait with a disease-allele frequency of 0.0001 and a penetrance of 99%. The allele frequencies for each marker were assumed to be equal, as well as the recombination frequencies in men and women. In the calculations, individuals II:1, III:1, III:2, III:7, III:8, III:9, IV:1, IV:2, IV:3, and IV:4 of kindred MF1 and I:1, II:2, II:3, III:1, and III:2 of kindred MF2 were scored as affected and all other members as unaffected. Family and haplotype data were generated using Cyrillic, Version 2.1 software.

### Deoxyribonucleic acid sequencing and population studies

The *FBN1* gene was screened for mutations with Sanger sequencing of all 66 exons and flanking splice sites. The consensus coding sequence of *FBN1* (CCDS32232) downloaded from NCBI build 36.2 was used as the reference sequence for the mutation analysis. The oligonucleotide sequences are detailed in Appendix 1. After amplification, the PCR products were purified and sequenced using the ABI BigDye Terminator cycle sequencing kit v3.1 (Applied Biosystems), according to the manufacturer’s instructions. Sequencing in both directions was performed on DNA samples from two affected individuals (III:1 and III:7 of MF1, II:2 and II:3 of MF2) and one unaffected individual (III:3 of MF1, II:1 of MF2) in each family. The sequencing products were run and analyzed using the ABI 3730 Genetic Analyzer (Applied Biosystems). Exons with detected variations were sequenced in all family members to evaluate whether the variations represent disease-association mutations. One hundred unrelated control individuals were tested for the identified mutations by using direct sequencing to determine whether they could be considered recurrent mutations or polymorphisms, and to confirm their association with the pathologic condition under study.

### Statistical analysis

An unpaired Student *t* test was performed to compare the aortic root diameters between the MF1 group (the affected individuals over 18 from MF1 family, n=10) and the MF2 group (the affected individuals over 18 from MF2 family, n=3), and between the MF1 group and controls (ages range from 30 to 50, n=5). p<0.05 was considered significant. Data in figures represent mean±standard deviation (SD) .

## Results

### Clinical evaluations

**Figure 2:**
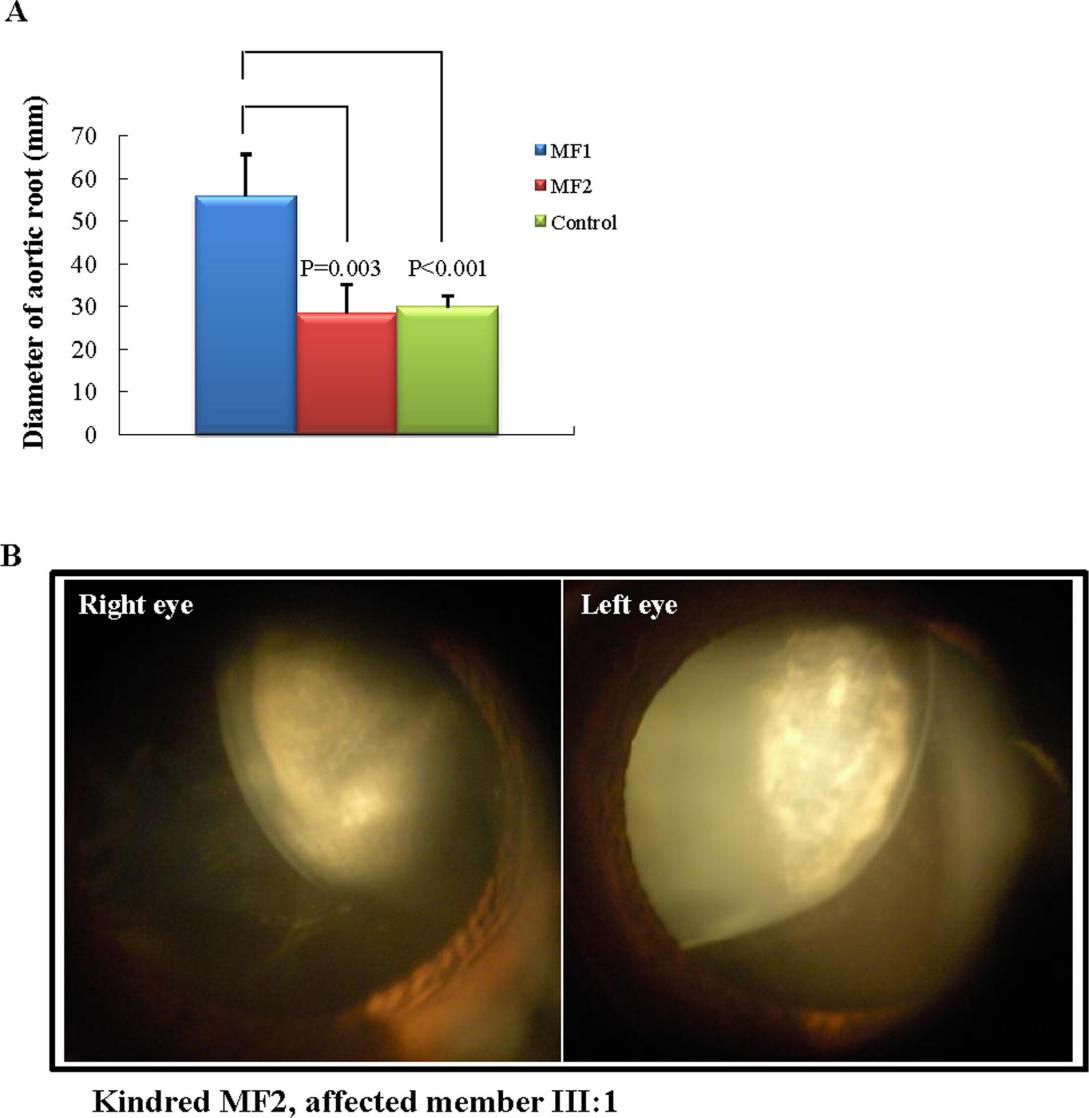
Clinical examination of two families. **A**: Comparisons for aortic root diameter measured with ultrasound examinations revealed significant differences between group MF1 (n=6) and group MF2 (n=3), and between group MF1 and controls (n=5). Error bars represent standard deviation. **B**: Ophthalmic examination of anterior segments shows a bilateral lens dislocation in individual III:1 of kindred MF2.

All 15 patients from both families (kindred MF1 and MF2) were clinically evaluated, and the two families were compared. According to the clinical findings schematically outlined in Table 1, the MF1 family has more severe systemic involvement than the MF2 family does. Notably, the ultrasonic examination revealed that the adult affected members in the MF1 family had a significantly increased diameter of the aortic root compared with age-matched controls (p<0.001), and compared with the adult affected members in the MF2 family (p=0.003; Table 1, Figure 2A). Moreover, the skeletal system was commonly and severely involved in patients in the MF1 family, whereas patients in the MF2 family mainly manifest increased height and arm span, and only one patient has joint hypermobility (Table 1). Unlike the systemic involvements, bilateral lens dislocation was observed in all affected members in the MF2 family (Figure 2B), but only in two out of ten patients in the MF1 family (Table 1).

### Linkage analysis

The only two known loci related to MFS were initially screened with nine microsatellite markers (D15S659, D15S992, D15S1016, D15S648, D3S2336, D3S2335, D3S1283, D3S3727, and D3S2432). After the *TGFBR2* locus on chromosome 3 was excluded (data not shown), positive linkage was found with markers of the *FBN1* locus in the 15q21.1 region in both families. A maximum multipoint limit of detection score of 2.83 was obtained for marker D15S992 in the MF1 family, and 1.51 for the same marker in the MF2 family (Table 2). A haplotype between D15S659 and D15S648 was cosegregated with disease phenotype in each family (Figure 1A). Because D15S992 is located within the *FBN1* gene, our genetic analysis of both families then turned to mutation analysis of *FBN1*.

**Table 2:** Linkage between MFS and markers on 15q21–22 in MF1 and MF2 families

Marker	Position on Chr. 15	LOD Score (alpha=1.0)	
	CM	MF1	MF2
**D15S659**	44.1601	2.764	1.504
	45.1438	2.788	1.504
	46.6194	2.825	1.505
**D15S992**	46.6196	2.825	1.505
	48.0296	2.822	1.504
	49.8296	2.821	1.504
	51.3196	2.819	1.504
**D15S1016**	51.3198	2.819	1.504
	53.2395	2.641	1.504
	55.1593	2.512	1.504
**D15S648**	55.1595	2.512	1.504
	60.1594	2.282	1.378

### Mutation analysis

With direct sequencing of *FBN1*, one novel mutation was identified in each of the two studied families. In the MF1 family, a heterozygous base substitution, c.813C>A, was detected in exon 8. This single base substitution generates a premature stop codon (TGA) instead of a cysteine (TGC) at codon 271 (p.C271*). In the MF2 family, a heterozygous variation, c.1910G>A was found in exon 16, resulting in a cysteine substitution by tyrosine at codon 637 (p.C637Y). These two allelic variations were cosegregated with the disease phenotypes in the two families, respectively, and were absent in 200 chromosomes from 100 unrelated control individuals, probably excluding the possibility of a rare polymorphism.

### Discussion

Since the first causative *FBN1* mutation was reported in MFS [2], Over thousand of mutations have been described (HGMD). These mutations are spread throughout the gene without obvious predilection for any given region [9]. According to Robinson et al., causation could be suggested by the type of mutation: involvement of a highly conserved cysteine residue, nonsense, frameshift, or splice site mutation [10]. For instance, several cysteine substitutions in the *FBN1* gene were correlated with ectopia lentis as the sole or predominant manifestation [11]. In this study, we report the identification of two novel mutations in the *FBN1* gene, including one nonsense mutation leading to a premature termination codon (PTC) and one missense mutation resulting in the substitution of a cysteine residue. The two types of mutation correlate with distinct phenotypes observed in two families with MFS. Further evidence of the mutations’ causality is suggested by the evidence that both mutations segregated with the disease phenotypes in two families whereas neither was found in 200 chromosomes from 100 unrelated control individuals.

The MF1 family displayed rather severe involvement of the organ system but with significant intrafamilial phenotypic variability (Table 1). All adult patients in the MF1 family exhibited remarkable cardiovascular system phenotypes, and the proband’s brother (III:4) died of aortic dissection at the age of 30 years. All patients presented various skeletal phenotypes, but interestingly, only two patients had lens dislocation. The rather severe cardiovascular and skeletal system phenotypes with less occurrence of lens dislocation observed in the MF1 family were linked to a PTC mutation (c.813C>A) of the *FBN1* gene. PTC mutations predict the production of truncated fibrillin 1 monomers, which could interfere with the assembly of normal monomers into extracellular microfibrils [12]. However, the existence of putative truncated proteins has not been proved due to the possibility of nonsense-mediate decay of the mutant mRNA transcript. As the transcripts harboring PTC could be detected by the mRNA quality control system and eliminated before translation by nonsense-mediate decay (NMD), therefore, the heterozygous mutation c.813C>A of *FBN1* could cause haploinsufficiency of fibrillin 1 by 50%, which in turn results in more severe systemic involvement in the MF1 family. If this is the case, it would potentially suggest that loss of fibrillin 1 function by 50% can lead to remarkable phenotypes in multiple organs involving the cardiovascular and skeletal systems. Nevertheless, the mechanism by which PTC mutations exert an effect on extracellular fibrillin monomers is not yet clear.

Patients in the MF2 family had relatively mild phenotypes compared with those carried by the MF1 family. The MF2 family mainly manifested lens dislocation, with some mild symptoms of the skeletal system, but no obvious cardiovascular features of MFS. This mild phenotype was the consequence of a missense mutation (c.1910G>A) of the *FBN1* gene. This mutation is located in cb-EGF modules and substituted a conserved cysteine residue potentially implicated in disulfide bonding. As three disulfide bonds are required to maintain cb-EGF module-fold, the mutation would likely disrupt one of the three disulfides bonds of the affected cb-EGF modules and probably cause domain misfolding with secondary deleterious effects on the global structure of fibrillin or microfibrils [13]. However, the effects of this mutation at the protein level are complicated: Impaired trafficking [14], delayed secretion [15], and enhanced protease susceptibility [16] have been described.

At the phenotypic level, our study showed that patients with MFS with the PTC mutation (the MF1 family) had more common aortic dilatation, more striking skeletal features, and large joint laxity, coupled with a much lower risk of serious ocular manifestations. However, the cysteine substitution mutation in the MF2 family causes a mild phenotype of MFS lacking aortic dilatation, but with major involvement of the ocular system and mild skeletal manifestations. Similar correlations have been suggested previously [12,15,17], and these data collectively emphasize the importance of these correlations referring to the clinical consequence of specific types of *FBN1* mutations.

In general, FBN1 mutations have been associated with a broad spectrum of phenotypes, ranging from single connective tissue manifestations to lethal neonatal MFS [18,19]. The intrafamilial variability shown in the MF1 family also confirmed that the allelic mutation was not the only determinant of clinical severity; circumstances and other modified factors might contribute to the expression of a certain mutation as well.

Our results further expand the spectrum of FBN1 mutations, and, perhaps more importantly, confirm the potential relationship between mutation type and phenotype. This insight about these relationships will allow more precise assessment of the clinical diagnosis, management, prognosis at early stage [20], and genetic counseling. Moreover, this insight may potentially lead to tailor-made patient follow-up.

## Appendix 1. Primers for mutation screening of FBN1.

To access the data, click or select the words “Appendix 1.”
